# Audit of Physical Health Monitoring in Patients With Intellectual Disabilities Prescribed Antipsychotics in Adult Intellectual Disability Service in Rivington Unit, Greater Manchester Mental Health NHSFoundation Trust

**DOI:** 10.1192/bjo.2026.11727

**Published:** 2026-06-30

**Authors:** Ehab Elkorashy, Rupa Gupta

**Affiliations:** GMMH, Manchester, United Kingdom

## Abstract

**Aims:**

This audit aimed to:

Evaluate compliance with NICE and GMMH guidelines for physical health monitoring in adults with intellectual disabilities prescribed atypical antipsychotics.

- Identify gaps in current monitoring practices.

- Enhance patient safety by highlighting areas requiring improvement.Support better long-term outcomes through early detection of physical health issues.

**Methods:**

Electronic clinical records (Paris) were reviewed using a bespoke audit tool based on NICE and GMMH protocols. Data were collected for the period February 2024–January 2025. Variables included demographic information, intellectual disability severity, diagnosis, and completion of recommended physical health checks. Data were analysed descriptively using Excel and presented graphically.

**Results:**

Seventy-eight patients were included (approx. 60% of the caseload). Of these, 57.5% were male and 73.08% were White British. Most were aged 18–39 years, and around half had mild intellectual disability.Compliance with routine blood monitoring ranged from 71.79% to 76.92%. In contrast, prolactin monitoring was completed in only 28.21% of patients, and ECG completion was notably low at 17.95%. These findings highlight significant variation in adherence to different components of the monitoring protocol.

**Conclusion:**

While compliance with routine blood tests was generally good, prolactin andECG monitoring were markedly poor. Improved documentation, clearer communication with primary care, and targeted interventions are required to ensure comprehensive physical health monitoring for this vulnerable population.

